# The impact of long non-coding RNA *HOTTIP* genetic variants on oral cancer progression and clinicopathological characteristics

**DOI:** 10.7150/jca.117916

**Published:** 2025-08-11

**Authors:** Ping-Ju Chen, Chiao-Wen Lin, Chun-Wen Su, Yung-Ching Wang, Heng-Hsiung Wu, Hsiao-Ju Chu, Shun-Fa Yang, Shih-Chi Su, Cheng-Chen Huang, Ying-Erh Chou

**Affiliations:** 1 Department of Dentistry, Changhua Christian Hospital, Changhua, Taiwan.; 2 Department of Post-Baccalaureate Medicine, College of Medicine, National Chung Hsing University, Taichung, Taiwan.; 3 Institute of Medicine, Chung Shan Medical University, Taichung, Taiwan.; 4 Institute of Oral Sciences, Chung Shan Medical University, Taichung, Taiwan.; 5 Department of Dentistry, Chung Shan Medical University Hospital, Taichung, Taiwan.; 6 Department of Medical Research, Chung Shan Medical University Hospital, Taichung, Taiwan.; 7 Program for Cancer Biology and Drug Discovery, China Medical University, Taichung, Taiwan.; 8 Whole-Genome Research Core Laboratory of Human Diseases, Chang Gung Memorial Hospital, Keelung, Taiwan.; 9 Department of Medical Biotechnology and Laboratory Science, College of Medicine, Chang Gung University, Taoyuan, Taiwan.; 10 School of Medicine, Chung Shan Medical University, Taichung, Taiwan.; 11 Department of Otolaryngology, Chung Shan Medical University Hospital, Taichung, Taiwan.

**Keywords:** oral cancer, HOTTIP, polymorphism

## Abstract

Oral cancer is the sixth leading cause of cancer-related mortality worldwide. Recent studies suggest that long non-coding RNAs (lncRNAs) HOXA transcript at the distal Tip (HOTTIP) may influence oral cancer cell growth and invasion, but comprehensive genetic association studies evaluating the impact of *HOTTIP* single-nucleotide polymorphisms (SNPs) on oral cancer susceptibility, and clinicopathological features are lacking. In this study, we investigated the associations between SNPs in the *HOTTIP* gene and both oral cancer susceptibility and clinicopathological characteristics. A total of 1,192 controls and 1,205 oral cancer patients were genotyped for four *HOTTIP* SNPs—rs3807598, rs17501292, rs2067087, and rs1859168—using real-time polymerase chain reaction (PCR). Our results showed that among oral cancer patients aged 60 years or older, those carrying the *HOTTIP* rs3807598 "GC+CC" genotype had a significantly reduced risk of developing advanced clinical stage and lymph node metastasis. Additionally, carriers of the rs2067087 "CG+GG" polymorphic variants were associated with a lower risk of developing advanced clinical stages. In conclusion, our findings suggest that the *HOTTIP* rs3807598 and rs2067087 polymorphisms may serve as pivotal predictor for assessing oral cancer progression.

## Introduction

Oral cancer is one of the most common cancers worldwide especially among males 1, 2. In Taiwan, oral cavity and oropharynx cancers together rank sixth place in cancer incidence and the fifth place in males 3. Tobacco smoking and smokeless tobacco use (direct or indirect exposure to tobacco products), heavy alcohol consumption, and betel quid chewing were suggested to be primary carcinogenic risk factors to oral cancer 4-6.

Long non-coding RNAs (lncRNAs) HOXA transcript at the distal Tip (HOTTIP) is located on human chromosome 7q15.2, and it is transcribed from the antisense strand at the 5' end of the HOXA gene cluster 7-9. HOTTIP is mainly binds to the WDR5/MLL complex, driving histone H3 lysine 4 trimethylation and the transcriptional activation of the terminal gene HOXA to upregulate the expression of development-related genes 7, 10.

HOTTIP has been shown to promote cancer cell proliferation, invasion, epithelial-mesenchymal transition (EMT), and metastasis in several malignancies such as ovarian cancer, breast cancer, hepatocellular carcinoma and oral cancer 11-20. For example, Li et al. reported that HOTTIP promotes oral cancer cell proliferation and migration by modulating microRNA-206 16. Moreover, Xiong et al. suggest that HOTTIP may act as an oncogene, contributing to oral cancer progression by miR-124-3p/HMGA2 axis through Wnt/β-catenin pathway 21. Additionally, the *HOTTIP* single-nucleotide polymorphisms (SNPs) were indicated to be associated with cancer development and prognosis in various cancers such as breast cancer (BC) 22, colorectal cancer (CRC) 23, 24, gastric cancer (GC) 25, 26, gastrointestinal (GI) cancers 27, hepatocellular carcinoma (HCC) 28, lung cancer 29, neuroblastoma 30, and pancreatic cancer 31. However, the associations of *HOTTIP* polymorphisms to oral cancer progression and clinicopathologic characteristics remained unclear. In this study, we examined four SNPs of *HOTTIP* rs3807598, rs17501292, rs2067087, and rs1859168, and try to elucidate their correlations to oral cancer susceptibility and clinicopathologic characteristics with environmental risk factors.

## Materials and Methods

### Study subjects

A total of 1205 male oral cancer patients and 1192 cancer-free controls were participated in our study. All the participants were recruited at Chung Shan Medical University Hospital, Taichung, Taiwan. For the demographic data, the age and gender were reported by each participant. The control group who enrolled in our study was those individuals who without self-reported diseases such as history of cancer of any sites. The informed written consent was provided to each patient who enrolled in this study. This project was approved by the institutional review board of Chung Shan Medical University Hospital (CSMUH No: CS1-21151).

### Sample preparation and DNA extraction

For genomic DNA extraction, we collected the peripheral blood specimens from normal controls and oral cancer patients who participated in our study 32. Each peripheral whole blood samples were preserved with EDTA containing tubes. The samples of peripheral whole blood were centrifuged under the settings of 3000 rpm, 10 minutes. The centrifuged buffy coats from the whole blood specimens were collected and further used to extract DNA. To acquire the DNA, the genomic DNA extraction assay was performed under the manufacturer's manual of QIAamp DNA blood mini kits. The DNA elution was completed with the Tris-EDTA (TE) buffer. Extracted DNA was applied for DNA template in further real-time polymerase chain reactions (PCRs).

### Selection of *HOTTIP* SNPs

In our study, we selected four *HOTTIP* SNPs rs3807598, rs17501292, rs2067087, and rs1859168 based on the International HapMap Project database 33. The SNP rs3807598 was included because previous studies have suggested that the *HOTTIP* rs3807598 polymorphism is associated with an increased risk of colorectal cancer (CRC) 24 and GC risk 26. The rs17501292 variant was selected due to evidence indicating that it may improve overall survival (OS) in CRC patients, particularly in the ulcerative/invasive-type tumor subgroup 24. The rs2067087 polymorphism was chosen because it has been reported to be associated with an elevated risk of CRC 24, increased susceptibility to HCC 28, and a potential link to GC risk 26. Finally, rs1859168 was selected because the C > A polymorphism has been associated with reduced neuroblastoma susceptibility in Chinese children 30, while the CC genotype and C allele have been linked to increased BC risk 22. Moreover, expression quantitative trait locus (eQTL) analysis revealed that the rs1859168 CC genotype was related to high expression of the HOTTIP gene of neuroblastoma in Chinese children 30. Wang et al. reported that the rs2067087 and rs3807598 SNPs of HOTTIP are associated with gastric cancer risk, possibly by affecting the expression of mature HOTTIP 26.

### *HOTTIP* SNPs genotyping

Assessment of allelic discrimination for the *HOTTIP* rs3807598 (assay IDs: C__30343054_10), rs17501292 (assay IDs: C__27835598_10), rs2067087 (assay IDs: C__15951120_10), and rs1859168 (assay IDs: C__11173652_30) SNP was performed with an ABI StepOne Software v2.3 Real-Time PCR System. The allelic discrimination for the four selected loci was evaluated using the TaqMan® SNP Genotyping Assay on an ABI StepOne™ Real-Time PCR System (Applied Biosystems, Foster City, CA, USA) 34, 35. Each 20 µL PCR reaction contained 10 µL of TaqMan® Genotyping Master Mix, 1 µL of 20× TaqMan® SNP Genotyping Assay Mix (containing allele-specific VIC® and FAM™ labeled probes and locus-specific primers), 10 ng of genomic DNA template, and nuclease-free water to adjust the final volume. The thermal cycling conditions were as follows: an initial denaturation step at 95°C for 10 minutes, followed by 40 cycles of denaturation at 95°C for 15 seconds and annealing/extension at 60°C for 1 minute. The analysis and calculation of the collected data of genotyping was processed with the SDS 7000 series software (Applied Biosystems, Foster City, CA, USA).

### Statistical analysis

The comparison of the age (years), betel quid chewing, cigarette smoking, alcohol drinking, tumor stage, tumor T status, lymph node status, metastasis, and cell differentiation between the oral cancer patients and the controls was evaluated with the student's t test or Chi-squared test. The association between the genotypic frequencies of *HOTTIP* and the clinicopathological features of oral cancer patients was assessed using multiple logistic regression models to calculate odds ratios (ORs) with 95% confidence intervals (CIs). A p < 0.05 was suggested to present statistically significant.

## Results

The distribution of demographical characteristics in 1192 controls and 1205 male patients with oral cancer was listed in Table 1. In the current study, we observed that the distributions of age (years) < 60 was 774 (64.9%) in controls and 740 (61.4%) in oral cancer patients, and the age ≥ 60 in controls and oral cancer patients was 418 (35.1%) and 465 (38.6%), respectively. The distributions of environmental risk factors exposure between the controls and oral cancer patients were 198 (16.6%) and 849 (70.5%) in betel quid chewing (p < 0.001), 634 (53.2%) and 970 (80.5%) in cigarette smoking (p < 0.001), and 235 (19.7%) and 495 (41.1%) in alcohol drinking (p < 0.001), respectively.

**Figure 1:**
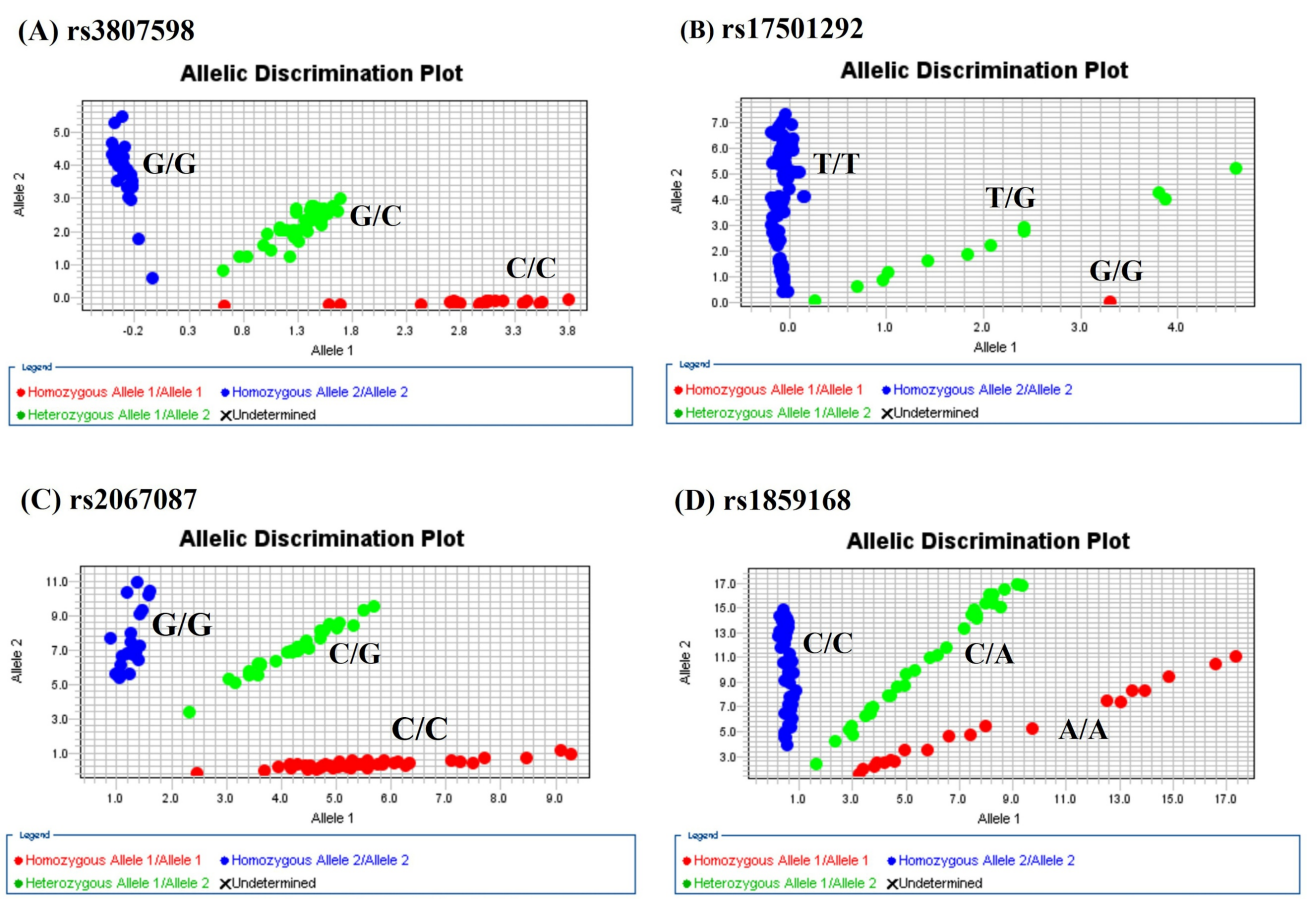
Allelic discrimination plot obtained for the HOTTIP SNP. (A) rs3807598, (B) rs17501292, (C) rs2067087, and (D) rs1859168 using a TaqMan assay. The x-and y-axes indicate the fluorescence values of the VIC and FAM dyes, respectively, while the dots are individual sample points.”

The genotype distributions of *HOTTIP* gene polymorphisms in 1192 controls and 1205 male patients with oral cancer were listed in Table 2. As shown in Figure 1, the most frequently occurring alleles were G/C for rs3807598, T/T for rs17501292, and C/G for rs2067087, C/A for rs1859168 (Figure 1). The adjusted odds ratios (AOR) with their 95% confidence intervals (CIs) were estimated by multiple logistic regression models. After adjustment for the effects of age, betel quid chewing, cigarette smoking, and alcohol drinking, no significant associations were found between the oral cancer patients and the controls (Table 2).

We further analyzed the odds ratio (OR) and 95% CIs of clinical statuses associated with genotypic frequencies of* HOTTIP* SNPs in male oral cancer patients. For *HOTTIP* rs3807598, no significant association was found between the *HOTTIP* rs3807598 polymorphisms and clinical statuses in oral cancer patients (Table 3). For *HOTTIP* rs2067087, in male oral cancer patients, a significant association was found between *HOTTIP* rs2067087 “CG+GG” genotype and metastasis [OR (95% CI): 0.115 (0.013-0.985); p = 0.017] (Table 4). Moreover, in male oral cancer patients with age ≥ 60, a significant association was found in those individuals who carried the *HOTTIP* rs3807598 polymorphic variant C, with a lower risk of clinical stage [OR (95% CI): 0.590 (0.393-0.886); p = 0.011], and lymph node metastasis [OR (95% CI): 0.649 (0.422-0.997); p = 0.047] (Table 5). Additionally, statistically significant association was found between the *HOTTIP* rs2067087 “CG+GG” polymorphic variants and clinical stage in male oral cancer patients with age ≥ 60 [OR (95% CI): 0.653 (0.444-0.962); p = 0.030] (Table 6). However, for *HOTTIP* 17501292 and rs1859168, no significant association was found between these SNPs in male oral cancer patients and patients with age ≥ 60 (data not shown).

## Discussion

In this study, we discovered the associations between the *HOTTIP* SNPs and oral cancer. Heavy alcohol consumption, betel quid chewing, and tobacco smoking are well-known risk factors responsible for oral cancer carcinogenesis, disease development, and progression 3, 36-38. About 90% of oral cancer was oral squamous cell carcinoma (OSCC) 16, 39. In our study, statistically significant associations of these risk factors including betel quid chewing, cigarette smoking, and alcohol drinking were found in 1205 male patients with oral cancer compared with 1192 controls, respectively. For the correlations of these risk factors to HOTTIP expression, a previous study has suggested that the lncRNAs HOTTIP was over expressed in extracellular vesicles (EVs) from smokers and NSCLC patients 40, and HOTTIP was found to be overexpressed in squamous cell carcinoma and in smokers 41. Therefore, although the information of alcohol consumption, betel quid chewing, and their synergistic effect combined with tobacco smoking to HOTTIP expression remained unclear till date, and the lncRNAs HOTTIP was suggested to be involved in disease development and progression, proliferation, migration, and invasion of oral cancer 15, 16, 42. It can be proposed that the tobacco smoking may play a vital role to influence the HOTTIP expression in OSCC patients.

We further examined the correlations of the *HOTTIP* genotypic frequencies to oral cancer susceptibility. Although these *HOTTIP* SNPs have not been previously investigated in oral cancer specifically, their known associations with cancer-related outcomes in other epithelial malignancies 22-26, 29, and the functional importance of HOTTIP in oral cancer biology suggest a plausible role in oral carcinogenesis. However, in our study, no statistically significant associations were found between the oral cancer patients and the controls, suggesting a limited disease susceptibility and carcinogenic effect of *HOTTIP* polymorphisms in oral cancer development. Intriguingly, after we analyzed clinical statuses associated with the *HOTTIP* genotypic frequencies among male oral cancer patients and patients with age ≥ 60, we found that the *HOTTIP* rs3807598 “GC+CC” polymorphic variants were associated with lower risk of clinical stage and lymph node metastasis, and the *HOTTIP* rs2067087 “CG+GG” genotype were associated with lower risk of metastasis in male oral cancer patients and lower risk of clinical stage in male oral cancer patients with age ≥ 60, respectively.

Generally, aging was considered as the most important risk factor of malignant disease and was suggested to be the largest risk factor for the development of cancer 43-45. Most cancers were found to arise in individuals over the age of 60 46. The ageing microenvironment may influence tumor progression 46, and molecular alterations in tumors may differ among patients of different ages 47. Compared with these results, in our study, the clinical statuses associated with genotypic frequencies of *HOTTIP* rs3807598 and rs2067087 polymorphisms were different in male oral cancer patients and patients with age ≥ 60, suggesting the possible change and influences of ageing microenvironment to tumor progression and molecular alterations with age in these oral cancer patients. For the correlations between the *HOTTIP* rs3807598 and rs2067087 to disease or cancer, some studies have associated the *HOTTIP* rs3807598 and rs2067087 with increased disease susceptibility and cancer risk such as CRC 24, and GC 26. However, in HCC, the *HOTTIP* rs2067087 was suggested to be associated with increased HCC risk, while the *HOTTIP* rs3807598 variant genotype was found to show significantly longer survival time in HBV negative subgroup, and no significant associations between the *HOTTIP* rs3807598 and HCC risk was found in the same study 28. Another study which focused on Hirschsprung disease has suggested a negative association between the lncRNAs *HOTTIP* rs3807598 C > G and risk of Hirschsprung disease 48. Taken together, although the lncRNAs HOTTIP has demonstrated to be associated with oncogenic regulation in cancer progression in various cancers including oral cancer 7, 8, 15, 16, 19, 26-28, 41, and the *HOTTIP* rs3807598 and rs2067087 were suggested to be associated with cancer risk 24, 26. However, the inconsistency of *HOTTIP* rs3807598 and rs2067087 to cancer risk, disease susceptibility, and survival time still observed 28, 48. One possible mechanism to explain these inconsistencies is that the HOTTIP was suggested to be functioned as a competing endogenous RNA (ceRNA). In renal cell carcinoma (RCC), it was reported that the HOTTIP down-regulation attenuated RCC cell proliferation, migration, and invasion, which could be rescued by miR-506 down-regulation 30. In acute myocardial infarction (AMI), HOTTIP knockdown markedly promoted cardiomyocyte growth and inhibited cardiomyocyte apoptosis in vitro, and miR-92a-2 overexpression could significantly enhance the protective effect of HOTTIP knockdown against AMI through partially inhibiting c-Met expression 49. Therefore, it might be the ageing microenvironment and the vital regulatory role of HOTTIP ceRNA which contribute to the better prognosis of *HOTTIP* rs3807598 and rs2067087 polymorphisms in male oral cancer patients with age ≥ 60 in our study. However, future well-designed studies are required to elucidate the detailed mechanisms and inconsistencies between the *HOTTIP* polymorphism in oral cancer and other diseases.

A key limitation of our study is the absence of HOTTIP expression data in cancer versus control samples. Due to the design of the current study, we were unable to collect tissue specimens or preserve whole-blood RNA for expression analysis. Nevertheless, previous studies have consistently shown that HOTTIP is significantly upregulated in oral cancer tissues compared to adjacent normal tissues, supporting its potential oncogenic role in oral cancer 50. Therefore, validating the clinical relevance of our genetic findings through expression profiling is warranted and will be a focus of future investigations.

In conclusion, our study first revealed the associations of *HOTTIP* polymorphisms to oral cancer disease susceptibility and clinical statuses. In male oral cancer patients with age ≥ 60, patients who carried the *HOTTIP* rs3807598 “GC+CC” genotype was associated with lower risk of clinical stage and lymph node metastasis, while carriers with *HOTTIP* rs2067087 polymorphic “CG + GG” genotype was associated with lower risk of clinical stage, respectively. The *HOTTIP* 3807598 and rs2067087 polymorphisms may provide as possible predictor to evaluate oral cancer disease progression and prognosis.

## Figures and Tables

**Table 1:** The distributions of demographical characteristics in 1192 controls and 1205 male patients with oral cancer.

Variable	Controls (N=1192)	Patients (N=1205)	p value
Age (yrs)			
< 60	774 (64.9%)	740 (61.4%)	p = 0.074
> 60	418 (35.1%)	465 (38.6%)	
Betel quid chewing			
No	994 (83.4%)	356 (29.5%)	
Yes	198 (16.6%)	849 (70.5%)	p < 0.001*
Cigarette smoking			
No	558 (46.8%)	235 (19.5%)	
Yes	634 (53.2%)	970 (80.5%)	p < 0.001*
Alcohol drinking			
No	957 (80.3%)	710 (58.9%)	
Yes	235 (19.7%)	495 (41.1%)	p < 0.001*
Stage			
I+II		552 (45.8%)	
III+IV		653 (54.2%)	
Tumor T status			
T1+T2		571 (47.4%)	
T3+T4		634 (52.6%)	
Lymph node status			
N0		813 (67.5%)	
N1+N2+N3		392 (32.5%)	
Metastasis			
M0		1199 (99.5%)	
M1		6 (0.5%)	
Cell differentiation			
Well differentiated		193 (16.0%)	
Moderately or poorly differentiated		1012 (84.0%)	

* p value < 0.05 as statistically significant.

**Table 2:** Odds ratio (OR) and 95% confidence interval (CI) of oral cancer associated with *HOTTIP* genotypic frequencies.

Variable	Controls (N=1192) (%)	Patients (N=1205) (%)	AOR (95% CI)	p value
**rs3807598**				
GG	302 (25.3%)	347 (28.8%)	1.000 (reference)	
GC	617 (51.8%)	571 (47.4%)	0.818 (0.651-1.029)	p=0.087
CC	273 (22.9%)	287 (23.8%)	0.950 (0.725-1.244)	p=0.707
GC+CC	890 (74.7%)	858 (71.2%)	0.858 (0.692-1.064)	p=0.163
**rs17501292**				
TT	1061 (89.0%)	1065 (88.4%)	1.000 (reference)	
TG	128 (10.7%)	136 (11.3%)	1.117 (0.824-1.515)	p=0.477
GG	3 (0.3%)	4 (0.3%)	2.139 (0.410-11.150)	p=0.367
TG+GG	131 (11.0%)	140 (11.6%)	1.122 (0.830-1.516)	p=0.456
**rs2067087**				
CC	430 (36.1%)	442 (36.7%)	1.000 (reference)	
CG	573 (48.1%)	511 (42.4%)	0.825 (0.666-1.021)	p=0.077
GG	189 (15.8%)	252 (20.9%)	1.251 (0.951-1.648)	p=0.110
CG+GG	762 (63.9%)	763 (63.3%)	0.945 (0.775-1.152)	p=0.578
**rs1859168**				
CC	432 (36.2%)	480 (39.8%)	1.000 (reference)	
CA	591 (49.6%)	533 (44.2%)	0.795 (0.628-1.018)	p=0.056
AA	169 (14.2%)	192 (16.0%)	1.064 (0.796-1.422)	p=0.676
CA+AA	760 (63.8%)	725 (60.2%)	0.836 (0.687-1.018)	p=0.075

The adjusted odds ratio (AOR) with their 95% confidence intervals were estimated by multiple logistic regression models after controlling for age, betel quid chewing, cigarette smoking, and alcohol drinking.

**Table 3:** Odds ratio (OR) and 95% confidence intervals (CI) of clinical statuses associated with genotypic frequencies of *HOTTIP* rs3807598 in male oral cancer patients.

Variable	GG (N=347)	GC+CC (N=858)	OR (95% CI)	p value
**Clinical Stage**				
Stage I+II	144 (41.5%)	408 (47.6%)	1.000 (reference)	p=0.056
Stage III+IV	203 (58.5%)	450 (52.4%)	0.782 (0.608-1.007)	
**Tumor size**				
≦ T2	165 (47.6%)	406 (47.3%)	1.000 (reference)	p=0.942
> T2	182 (52.4%)	452 (52.7%)	1.009 (0.786-1.296)	
**Lymph node metastasis**				
No	221 (63.7%)	592 (69.0%)	1.000 (reference)	p=0.075
Yes	126 (36.3%)	266 (31.0%)	0.788 (0.606-1.024)	
**Metastasis**				
M0	343 (98.8%)	856 (99.8%)	1.000 (reference)	p=0.061
M1	4 (1.2%)	2 (0.2%)	0.200 (0.037-1.099)	
**Cell differentiated grade**				
Well	51 (14.7%)	142 (16.6%)	1.000 (reference)	p=0.427
Moderate or poor	296 (85.3%)	716 (83.4%)	0.869 (0.614-1.230)	

The odds ratio (OR) with their 95% confidence intervals were estimated by logistic regression models.

**Table 4:** Odds ratio (OR) and 95% confidence intervals (CI) of clinical statuses associated with genotypic frequencies of *HOTTIP* rs2067087 in male oral cancer patients.

Variable	CC (N=442)	CG+GG (N=763)	OR (95% CI)	p value
**Clinical Stage**				
Stage I+II	190 (43.0%)	362 (47.4%)	1.000 (reference)	p=0.134
Stage III+IV	252 (57.0%)	401 (52.6%)	0.835 (0.767-1.037)	
**Tumor size**				
≦ T2	206 (46.6%)	365 (47.8%)	1.000 (reference)	p=0.680
> T2	236 (53.4%)	398 (52.2%)	0.952 (0.753-1.204)	
**Lymph node metastasis**				
No	298 (67.4%)	515 (67.5%)	1.000 (reference)	p=0.978
Yes	144 (32.6%)	248 (32.5%)	0.997 (0.776-1.280)	
**Metastasis**				
M0	437 (98.9%)	762 (99.9%)	1.000 (reference)	p=0.017*
M1	5 (1.1%)	1 (0.1%)	**0.115 (0.013-0.985)**	
**Cell differentiated grade**				
Well	77 (17.4%)	116 (15.2%)	1.000 (reference)	p=0.312
Moderate or poor	365 (82.6%)	647 (84.8%)	1.177 (0.858-1.613)	

The odds ratio (OR) with their 95% confidence intervals were estimated by logistic regression models. * p value < 0.05 as statistically significant.

**Table 5:** Odds ratio (OR) and 95% confidence intervals (CI) of clinical statuses associated with genotypic frequencies of *HOTTIP* rs3807598 in male oral cancer patients with age > 60.

Variable	GG (N=136)	GC+CC (N=329)	OR (95% CI)	p value
**Clinical Stage**				
Stage I+II	53 (39.0%)	171 (52.0%)	1.000 (reference)	p=0.011*
Stage III+IV	83 (61.0%)	158 (48.0%)	**0.590 (0.393-0.886)**	
**Tumor size**				
≦ T2	66 (48.5%)	159 (48.3%)	1.000 (reference)	p=0.969
> T2	70 (51.5%)	170 (51.7%)	1.008 (0.676-1.504)	
**Lymph node metastasis**				
No	88 (64.7%)	243 (73.9%)	1.000 (reference)	p=0.047*
Yes	48 (35.3%)	86 (26.1%)	**0.649 (0.422-0.997)**	
**Metastasis**				
M0	135 (99.3%)	328 (99.7%)	1.000 (reference)	p=0.518
M1	1 (0.7%)	1 (0.3%)	0.412 (0.026-6.628)	
**Cell differentiated grade**				
Well	22 (16.2%)	54 (16.4%)	1.000 (reference)	p=0.950
Moderate or poor	114 (83.8%)	275 (83.6%)	0.983 (0.572-1.689)	

The odds ratio (OR) with their 95% confidence intervals were estimated by logistic regression models. * p value < 0.05 as statistically significant.

**Table 6:** Odds ratio (OR) and 95% confidence intervals (CI) of clinical statuses associated with genotypic frequencies of *HOTTIP* rs2067087 in male oral cancer patients with age > 60.

Variable	CC (N=160)	CG+GG (N=305)	OR (95% CI)	p value
**Clinical Stage**				
Stage I+II	66 (41.3%)	158 (51.8%)	1.000 (reference)	p=0.030*
Stage III+IV	94 (58.8%)	147 (48.2%)	**0.653 (0.444-0.962)**	
**Tumor size**				
≦ T2	76 (47.5%)	149 (48.9%)	1.000 (reference)	p=0.782
> T2	84 (52.5%)	156 (51.1%)	0.947 (0.646-1.389)	
**Lymph node metastasis**				
No	107 (66.9%)	224 (73.4%)	1.000 (reference)	p=0.137
Yes	53 (33.1%)	81 (26.6%)	0.730 (0.482-1.107)	
**Metastasis**				
M0	159 (99.4%)	304 (99.7%)	1.000 (reference)	p=0.642
M1	1 (0.6%)	1 (0.3%)	0.523 (0.032-8.418)	
**Cell differentiated grade**				
Well	29 (18.1%)	47 (15.4%)	1.000 (reference)	p=0.452
Moderate or poor	131 (81.9%)	258 (84.6%)	1.215 (0.731-2.020)	

The odds ratio (OR) with their 95% confidence intervals were estimated by logistic regression models. * p value < 0.05 as statistically significant.
